# An Information Tool Incorporating Real-World Outcome Data for Women With Metastatic Breast Cancer Eligible for Treatment With a CDK4/6 Inhibitor: Development and Evaluation

**DOI:** 10.2196/73156

**Published:** 2026-07-30

**Authors:** Ellen G Engelhardt, Mariska Q N Hackert, Anne Vogelaar, Afke F de Jong, Cristina Guerrero Paez, Annette W G van der Velden, Christa Putker, Mariette J Agterof, Ewoudt M W van de Garde, Cornelia F van Uden-Kraan

**Affiliations:** 1 Santeon (Netherlands), Herculesplein 102, Utrecht, 3584 AA, The Netherlands; 2 Stichting Kijksluiter, Amsterdam, The Netherlands; 3 Borstkankervereniging Nederland, Utrecht, The Netherlands; 4 Department of Internal Medicine, Martini Ziekenhuis, Groningen, The Netherlands; 5 Department of Internal Medicine, St. Antonius Hospital, Utrecht/Nieuwegein, The Netherlands; 6 Department of Pharmacy, St. Antonius Hospital, Utrecht/Nieuwegein, The Netherlands; 7 Department of Pharmaceutical Sciences, Division of Pharmacoepidemiology and Clinical Pharmacology, Utrecht University, Utrecht, The Netherlands

**Keywords:** information tool, real world outcome data, CDK4/6 inhibitor, advanced breast cancer, value-based health care

## Abstract

**Background:**

Accelerating the transition to value-based health care (VBHC) is essential to ensure sustainable care delivery. VBHC maximizes patient value by optimizing outcomes, controlling costs, and leveraging data to improve quality and patient-doctor communication. Integrating real-world data in health care is important to achieve informed decisions, especially in palliative care, where choices are complex.

**Objective:**

This study described the development and pilot-testing of an information tool that incorporates real-world outcome data for women with metastatic breast cancer who are initiating CDK4/6 inhibitor treatment. Real-world insights are needed to better inform these patients, in particular because real-world outcomes are known to differ from trial results because of larger heterogeneity in patient characteristics, and differences in frequency of check-ups and handling side effects.

**Methods:**

We developed an information tool together with patient representatives and clinicians using a participatory development approach that consisted of five key steps: (1) establishment of a multidisciplinary steering group (n=10 steering group members), (2) mapping of the patient journey and patients’ needs through focus groups (n=9) and semistructured interviews (n=8), (3) extraction of real-world outcome data from electronic health records systems of 229 patients, (4) prototyping of the tool (n=10), and (5) pilot evaluation with the targeted patient population using semistructured interviews (n=38). We used qualitative analysis methods to analyze the focus group and interview data.

**Results:**

We developed a tool consisting of (1) a communication aid for use during doctor-patient consultations (ie, KIJKgesprek [Stichting Kijksluiter]) and (2) a 2-component companion app with informational videos for use at home, both incorporating real-world outcome data (ie, KIJKbericht and KIJKsluiter [Stichting Kijksluiter]). Participants valued the tool for its clarity and structured design, reporting that the outcome data reinforced their experiences and facilitated the setting of realistic expectations. However, some participants described the outcome data as overwhelming, underscoring the importance of careful framing and delivery. Preferences regarding the type, level of detail, and timing of information presentation varied among participants, highlighting the necessity of individualizing information tools to meet diverse informational needs.

**Conclusions:**

Most patients valued the inclusion of real-world outcome data in the information tool, although many found it challenging to process. Preferences for the type and presentation of information varied widely among individuals. Information tools incorporating outcome data have the potential to enhance patient understanding and support informed decision-making about care that they value most. However, these tools must be designed to allow for customization, ensuring they address individual informational needs and preferences effectively.

## Introduction

Given recent health care challenges, accelerating the transition towards value-based health care (VBHC) has become urgent [1]. VBHC aims to maximize value for patients by optimizing patient-relevant care outcomes, while preferably lowering or maintaining the same costs. Outcome data play a major role in doing so. On a group level, outcome data are increasingly used for quality improvement through benchmarking, that is, comparisons over time, between treatments, and across institutions. On an individual patient level, outcome data should be considered an essential component of patient information and communication. However, such use of outcome data is still not standard in everyday clinical practice [2-5]. In particular, real-world outcome data, collected in daily clinical practice, can be used to support shared decision-making (SDM). These data help patients weigh the pros and cons of treatment options available to them, in line with their own values and expectations. This likely increases patients’ satisfaction and treatment compliance [5,6].

SDM is widely recognized for supporting preference-sensitive decisions, particularly when multiple treatment options offer similar benefits or when opting out of treatment is viable, but outcomes remain uncertain [7]. In palliative care, patients have complex needs [8]; however, their desired involvement in decision-making is often not achieved, and their information needs, including their need for prognostic information, are not met [9-11]. They are often not informed about expected survival benefit or the option to refrain from treatment, while the survival benefit is often small and uncertain. Simultaneously, the burden of treatment is likely high in terms of side effects and frequent check-ups [12]. Furthermore, drug performance in clinical trials often differs significantly from results observed in real-world settings [2,13-15], which likely complicates decision-making and raises questions about the applicability of trial findings to broader real-world populations whose characteristics may differ from those of trial participants.

We aimed to deliver a proof-of-concept of including real-world outcome data in patient information and communication. Therefore, we conducted a case study in women with hormone receptor–positive (HR_pos_), human epidermal growth factor receptor 2–negative (HER2_neg_) metastatic breast cancer (BC). This type accounts for 65% of all BC cases worldwide [16], of which 3%‐12% are projected to have metastatic disease [17] with a 5-year survival rate of approximately 37% [16]. From this target group, we included women eligible to receive the CDK4/6 inhibitor palbociclib combined with endocrine therapy. This targeted therapy aims for disease control by slowing tumor growth, prolonging patients’ progression-free survival (PFS). In clinical practice, CDK4/6 inhibitors are most often offered in second- or later-line, following progression on previous endocrine therapy. By targeting cell cycle regulation, CDK4/6 inhibitors reverse or delay resistance to endocrine treatment, and delay the need for more burdensome chemotherapy. Clinical trials have reported promising results (eg, improved time until disease progression with 4‐10 months [18,19]); however, there are also data that outcomes differ in real-world settings. For example, how modifications are handled in case of neutropenia [20,21]. Based on these considerations, we prioritized the use of real-world data in this research.

In this study, we aimed to (1) systematically develop a patient information tool incorporating real-world outcome data to support patients in making informed decisions about treatment initiation and managing their care throughout the treatment process, and (2) evaluate the tool in a pilot study with the target population. We used a participatory development approach [22] to center this study on patients and health care professionals (HCPs), deepening our understanding of their needs and care experiences and enhancing the usefulness of our patient information tool for supporting SDM. In line with the VBHC emphasis on collaboration across patients, clinicians, organizations, and other stakeholders, participatory design brings these groups together in co-design. This helps surface challenges earlier and leads to more effective and user-friendly solutions.

## Methods

### Overview

**Figure 1.**
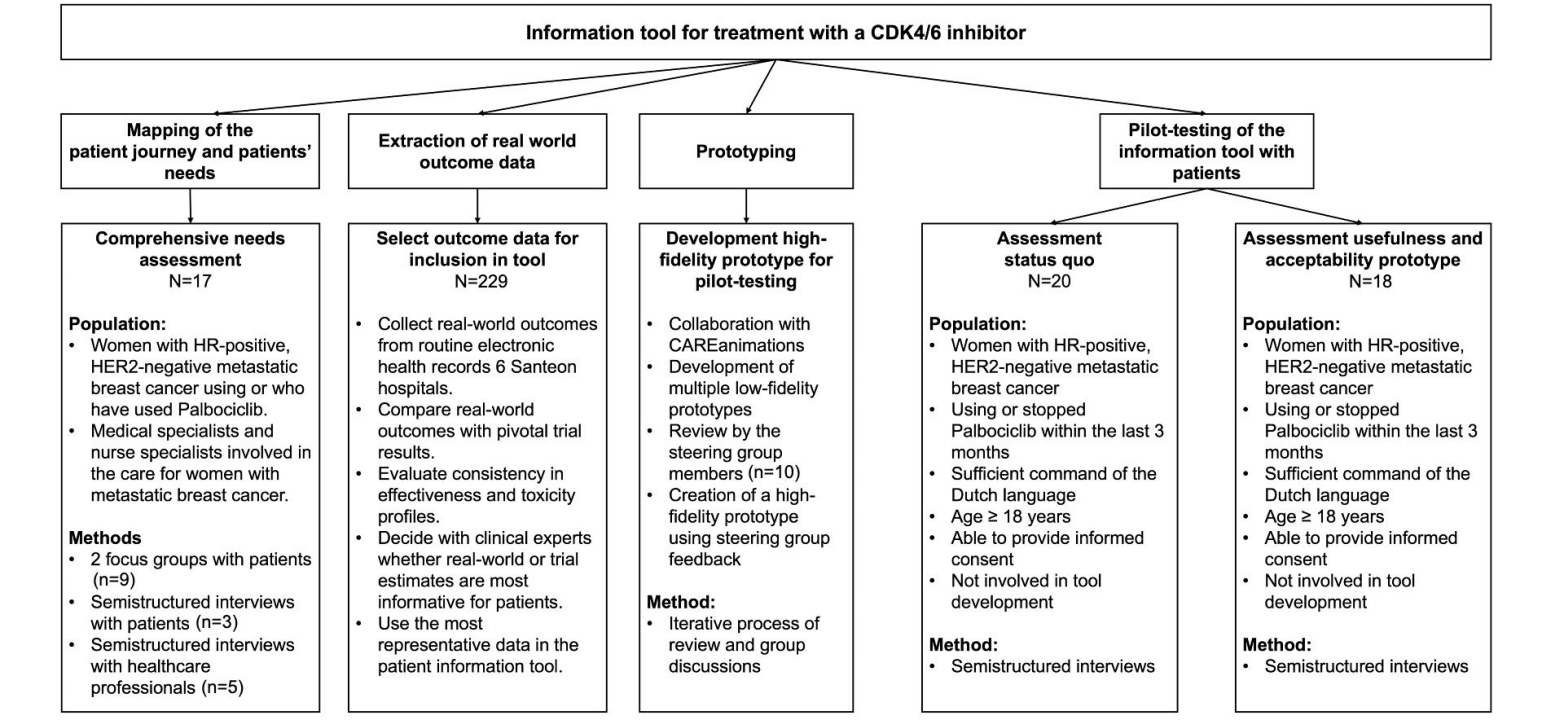
Flow chart development process components. HER2: human epidermal growth factor receptor 2; HR: hormone receptor.

We used qualitative methods to develop the CDK4/6 inhibitor real-world patient information tool. The development process consisted of five steps: (1) establishment of a multidisciplinary steering group, (2) mapping of the patient journey and patients’ needs through focus groups and semistructured interviews, (3) extraction of real-world outcome data from electronic health records systems, (4) prototyping of the tool, and (5) pilot evaluation with the targeted patient population using semistructured interviews (Figure 1).

### Ethical Considerations

The study protocol was approved by the Medical Research Ethics Committees United (Nieuwegein, the Netherlands; reference SDB2021-010). Ethics and research governance approval were obtained from the local medical ethics committees of the participating hospitals. Participating patients provided written informed consent.

### Establishment of a Multidisciplinary Steering Group

The development of the information tool was initiated by Santeon, a Dutch hospital group in which 7 top clinical hospitals openly collaborate to improve medical care [21]. The project initiators (postdoctoral researcher MQNH, program manager CFvUK, and hospital pharmacist EMWvdG) established a multidisciplinary steering group for the development of the tool.

### Mapping of the Patient Journey and Patients’ Needs

The patient journey mapping (ie, inventory of patients’ experiences from diagnosis until disease progression) served as the starting point in the development of the information tool. The goal was to obtain insight into patients’ experiences from diverse patients to help identify information and support needs that needed to be addressed in the patient information tool. We collaborated with the Patient Journey Lab, a company specialized in patient journey mapping, to develop the patient journey. First, to roughly outline the patient journey, 2 focus groups were conducted by MQNH and an independent research consultant (experienced in conducting focus groups) with 5 and 4 patients, respectively, that lasted about an hour. They focused on the following topics: (1) information provision, (2) decision-making to start the CDK4/6 inhibitor, (3) unmet information needs, and (4) perspectives on the use of real-world outcome data. Second, to verify and fill out the patient journey in greater detail, semistructured interviews, with an expected duration of 30 minutes, were conducted by MQNH with 3 patients and the 2 oncologists and 3 nurse specialists who participated in the steering group. All recordings were transcribed and summarized by MQNH. Manual summative content analysis [23] was used to infer meaning from recurring keywords and messages. Based on the topic list, keywords were selected to highlight in the text to count their frequencies before defining and contextualizing them in our final summary.

### Extraction of Real-World Outcome Data

To inform new patients about the treatment, the information tool includes outcome data of 229 patients treated between 2016 and 2020 in 6 Santeon hospitals (St Antonius Hospital Utrecht/Nieuwegein, Martini Hospital Groningen, Canisius-Wilhelmina Hospital Nijmegen, Catharina Hospital Eindhoven, OLVG Amsterdam, and Maasstad Hospital Rotterdam). More detailed information on the real-world cohort and the data collection has been reported previously and is available elsewhere [24,25]. Briefly, the data collected included PFS, the reason for ending treatment, the severity, frequency, and timing of all side effects and toxicities (with a special focus on neutropenia), and the timing and frequency of all treatment modification strategies. They were derived from routinely collected electronic health records, with 1 hospital excluded due to incomplete documentation of palbociclib use. To ensure the robustness of the real-world data to be included in the tool, we compared in this dataset outcomes in a cohort of 229 patients with HR_pos_, HER2_neg_ metastatic BC treated with palbociclib plus fulvestrant in second- or later-line therapy to those reported in the pivotal *PALOMA-3* trial that formed the basis for regulatory approval of palbociclib in this setting. On the basis of the comparison, the expert work group concluded that given the greater clinical heterogeneity and the mismatch in toxicity profiles compared with trial participants, real-world outcome estimates were more representative for informing patients and were therefore used in the information tool. [24,25]

### Prototyping

In consultation with the steering group and based on the patient journey mapping, it was decided to develop a digital information tool to allow future efforts to link the tool to patients’ electronic health records. Such a linkage provides two main benefits: (1) it gives patients access to all medical information in one place, and (2) it allows the tool to display up-to-date real-world outcome data based on automatic updates retrieved from personal data stored in electronic health record systems. To examine how the tool could best be integrated in the digital landscape of the Santeon hospitals, the Patient Journey Lab inventoried the software potential per hospital, including available health applications. As CAREanimations offers accessible information suitable for most people, including those with low (health) literacy, tailored to each step in the patient journey, it was selected as the development and implementation partner [26]. In collaboration with CAREanimations, the initiators developed several low-fidelity prototypes, which were reviewed by the steering group to arrive at a high-fidelity prototype for pilot-testing.

### Pilot-Testing of the Information Tool Among the Targeted Population

We carried out a pilot evaluation in 2 steps. First, before implementing the information tool, we assessed the status quo, that is, patients’ experiences with treatment and their met and unmet information and support needs. We did this to validate the findings of our previous unmet need assessment. Second, we assessed patients’ views on the usefulness and acceptability of the high-fidelity prototype of the information tool and inventoried suggestions for improvement.

In the first step, we conducted in-depth semistructured interviews with women with HR_pos_HER2_neg_ metastatic BC who (1) were using or had stopped using the CDK4/6 inhibitor Palbociclib within the last 3 months, (2) had sufficient command of the Dutch language, (3) were 18 years or older, (4) did not have (severe) cognitive impairment that would preclude them from providing informed consent, and (5) had not been involved in the development process. We obtained insights into patients’ (1) experiences with information provision about their diagnosis, prognosis, and treatment with CDK4/6 inhibitor; (2) experiences with treatment (eg, side effects and impact on daily functioning); (3) experiences with treatment decision-making; and (4) preferences regarding receipt of outcome data. In addition, we obtained information about sociodemographic characteristics (age and educational level) and medical history (date of primary BC diagnosis, treatment history; refer to Multimedia Appendix 1 for the full interview guide). We recruited participants from 5 Santeon hospitals (ie, St Antonius Hospital [Utrecht/Nieuwegein], Martini Hospital [Groningen], Canisius-Wilhelmina Hospital [Nijmegen], Catharina Hospital [Eindhoven], and Maasstad Hospital [Rotterdam]). Patients were invited to participate by their oncologist or nurse specialist. The interviews were conducted by EGE (researcher with interviewing experience).

In the second step, we evaluated the prototype of the information tool through individual semistructured interviews with a new cohort of women not previously involved in the development or pilot-testing, meeting the same inclusion criteria as described in step 1. Participants were recruited at the same 5 Santeon hospitals and via the Dutch Breast Cancer Society. Recruitment through the Dutch Breast Cancer Society broadened the target population, as participants were also treated at other hospitals, which accelerated the recruitment process. Two weeks before the interview, participants were provided with access to all the animation videos contained in the information tool and the graphical information intended for use during the consultation with their oncologist. They were asked to view all the materials and take notes of any suggestions for possible improvements. After they reviewed the information, a telephone interview was conducted by MQNH or EGE. The interview guide to assess patients’ experiences with treatment and their met and unmet information and support needs was used and extended to also obtain insight into patients’ views on the completeness and clarity of the CDK4/6 inhibitor information tool and to collect suggestions for improvement (refer to Multimedia Appendix 1 for the full interview guide).

All interviews (in the first and second steps) were audio-recorded with the participant’s permission and transcribed. The transcripts were analyzed using a combined technique of deductive and inductive thematic analysis [27,28]. For the deductive approach, we constructed an analytic framework based on predefined themes in the interview guide. In addition, we conducted open coding (inductive) on the transcripts to allow the inclusion of other relevant themes. We generated categories and abstractions through detailed review, quantifying keywords and phrases, and comparing findings. As educational level was considered to be an important predictor of patients’ comprehension of and satisfaction with the information tool, we explored differences in participants’ responses based on their educational background. All transcripts were coded by 2 researchers (MQNH and EGE) independently. Discrepancies were resolved through consensus. Qualitative data analyses were performed using Atlas.ti (version 9; Lumivero) software. Quotes are provided to illustrate our findings.

## Results

### Establishment of a Multidisciplinary Steering Group

**Figure 2.**
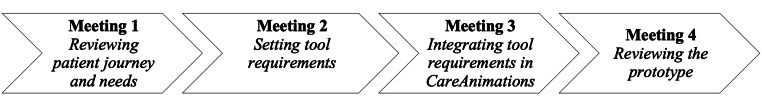
Focus of the cocreative steering group sessions.

The steering group consisted of patient representatives or advocates from the Dutch Breast Cancer Society (n=3), oncologists (n=2), specialist nurses (n=3), an information architect (n=1), and a health information consultant (n=1; for more details, refer to Multimedia Appendix 2). The steering group convened for 4 cocreation sessions. Figure 2 provides an overview of the focus of each session. The steering group was involved throughout the development process.

### Mapping the Patient Journey and Patients’ Needs

**Figure 3.**
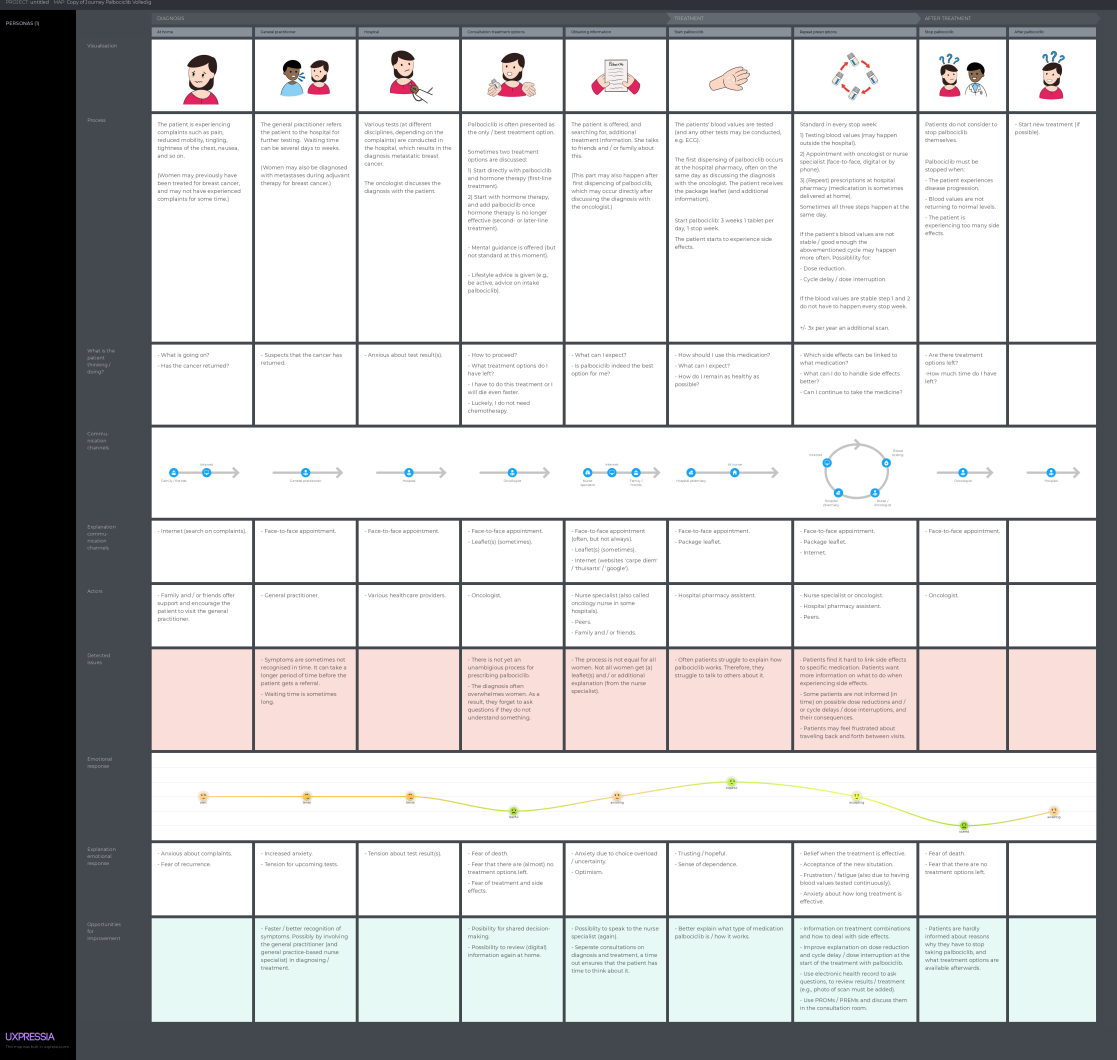
Detailed overview of the patient journey.

The findings from interviews and focus groups with patients (n=12) and clinicians (n=5) were used to map the patient journey of women undergoing treatment with a CDK4/6 inhibitor, from the start of treatment to its discontinuation (Table 1). A detailed overview of the patient journey is provided in Figure 3. Findings from the interviews with clinicians about the requirements for the information tool were in line with the findings from the patient interviews. All participants valued real-world outcome data in addition to clinical trial results, preferably displayed in a leaflet or app (connected to the electronic medical record). Information needed to be tailored to each step of the patient journey. Repetition and gradually offering more information were believed to be key. Even though dealing with a wide range and likely negative outcomes may be complex, participants wished insight into the timing, frequency, and severity of side effects, as well as the timing and reasons for ending treatment, so they could compare their outcomes to those of peers. Most patients wished to be better informed about possible medication alterations. Information on what type of treatment CDK4/6 inhibitor is, as well as what to do when experiencing side effects, was also valued. A summary of the key findings from the focus groups and interviews conducted to map the patient journey can be found in Table 2.

**Table 1.** Descriptive statistics of patients participating in focus groups and semistructured interviews (n=12).

Characteristics^a^	Patients
Age (y), range	38‐66
Missing, n	3
Education, n	
Low	1
Middle	4
High	5
Missing	2
Country of residence, n	
The Netherlands	10
Belgium	1
France	1
Breast cancer, n	
Metastatic recurrence	5
Immediate metastasis	6
Missing	1
Therapy, n	
First-line: palbociclib and letrozole or anastrozole	5
Second- or later-line: palbociclib and fulvestrant	6
Missing	1
Duration usage palbociclib, n	
Half a year or longer	12

a In a small number of interviews, certain sociodemographic questions were not asked or probed further because the conversation centered on highly emotional and sensitive topics, and the interviewer prioritized participant comfort and rapport.

**Table 2.** Summary of findings per theme and subtheme of the focus groups and interviews conducted to map the patient journey.

Theme and subtheme	Description
Information resources	
Health care professionals	Patients were informed about CDK4/6 inhibitor by their medical oncologist. After the initial consultation with their medical oncologist, some, but not all, patients received more detailed information from a nurse specialist or oncology nurse. Patients who did not have a dedicated nurse specialist or oncology nurse experienced a lack of guidance or support.
Websites and information leaflets	Some patients were referred to websites or information leaflets or were given an information leaflet at the hospital pharmacy. Patients liked receiving these additional information resources as the information received from health care providers was a lot to take in all at once, and these additional resources could serve as a reference for later.
Medication leaflet	All patients received the medication leaflet, which they handled differently. Some patients read it partly or in its entirety (multiple times) before starting with the treatment. Others consulted the medication leaflet upon experiencing complaints to avoid forgetting information, getting (unnecessarily) worried, or imagining symptoms that were not there. Some patients consulted peers on the internet.
Experiences with information provision about treatment	
Knowledge gap	To many patients it was not clear what type of treatment CDK4/6 inhibitor is: it was often mistaken for chemo- or immunotherapy. Patients used the internet to search for more detailed information on side effects and how to deal with them.
Variation in information provision and unmet information needs	Participants had mixed experiences with information provision about medication alterations (eg, lowering of the dosage or extension of the time interval between treatments). Some participants were informed about possible medication alterations before treatment started, some when it became relevant during treatment, whilst others were not consulted when an alteration was needed. Most patients wished to be more informed about their options.
Outcome data	
Preferences for receiving outcome data	Patients indicated they would value receiving real-world outcome data in addition to clinical trial results. Real-world outcome data, tailored to their medication combination, could help them compare their outcomes to those of peers, even though dealing with a wide range, and likely negative, outcomes may be complex.
Role in decision-making	
Oncologists	Upon diagnosis, the oncologist often immediately decided to start treatment with CDK4/6 inhibitor, as they believed it to be the best treatment option.
Patients	Some patients liked the oncologist to take the lead as they felt there was a need to act swiftly after their diagnosis and trusted their oncologist. Some patients were more involved in decision making, by proposing treatment with CDK4/6 inhibitor themselves, or by discussing alternatives (eg, chemotherapy) with their oncologist. Overall, conservative treatment was not considered an option by patients.
Requirements for the information tool	
Important content	A description of the type of treatment and what to do when experiencing side effects.
Preferences for incorporating outcome data in the information tool	Also, real-world outcome data related to the timing, frequency, and severity of side effects and medication alterations was deemed important to disclose in the tool, as well as the timing and reasons for ending treatment.
Type of tool and specifications	Patients liked to receive real-life outcome data in a leaflet, handed out by the nurse specialist or oncology nurse, but an app (connected to the electronic medical record) was also mentioned. In terms of information presentation, it was deemed important that the information in the tool was tailored to each step of the patient journey, and that repetition and gradually offering more detailed information is key.

### Extraction and Assessment of Real-World Outcome Data

Real-world outcome data were extracted from a cohort of patients treated with palbociclib in combination with fulvestrant in routine clinical practice. In this cohort, the median PFS was 11.6 (95% CI 10.2-13.9) months, which was longer than the 9.5 (95% CI 9.2-11.0) months reported for the PALOMA-3 trial population (more detailed information is available in the study by Hackert et al [24]). This difference may partly reflect differences in outcome assessment between clinical trials and routine clinical practice. When the real-world cohort was stratified according to PALOMA-3 eligibility criteria, differences in outcomes were observed. Patients who would have met the PALOMA-3 eligibility criteria had a median PFS of 14.1 (95% CI 11.5-17.0) months, whereas patients who would not have met these criteria had a median PFS of 10.2 (95% CI 8.8-12.3) months.

The occurrence of hematological toxicities and nonhematological side effects associated with palbociclib was generally similar in real-world settings compared with the clinical trial data. However, patients in routine clinical practice reported certain side effects more frequently, particularly fatigue and decreased appetite. In clinical practice, 54% (124/229) of patients experienced at least 1 cycle delay, 39% (89/229) had at least 1 dose reduction, and 26% (59/229) had at least 1 dose interruption, compared with 36% (124/345), 34% (117/345), and 54% (186/345), respectively, in the PALOMA-3 trial. Detailed findings on patient-reported experiences are described in the study by Vaisson et al [22].

### Choices in Data Presentation in the Information Tool

To support the development of the information tool, the extracted real-world outcomes were summarized using lay terminology and reviewed together with the steering group. Based on the extracted outcome data, decisions were made in collaboration with the steering group regarding which outcomes to include in the tool and how these should be presented to patients. Particular attention was given to ensuring that the translation of clinical outcomes into patient-friendly information accurately reflected the limitations of real-world data, such as potential selection bias, missing or incomplete data, and variability in outcome measurement and reporting. For example, rather than presenting outcome distributions based solely on real-world data, outcomes from clinical trial data and real-world data were compared, and presentation choices were made based on this comparison. This approach aimed to provide balanced and transparent information for patients using the information tool.

### Prototyping

**Figure 4.**
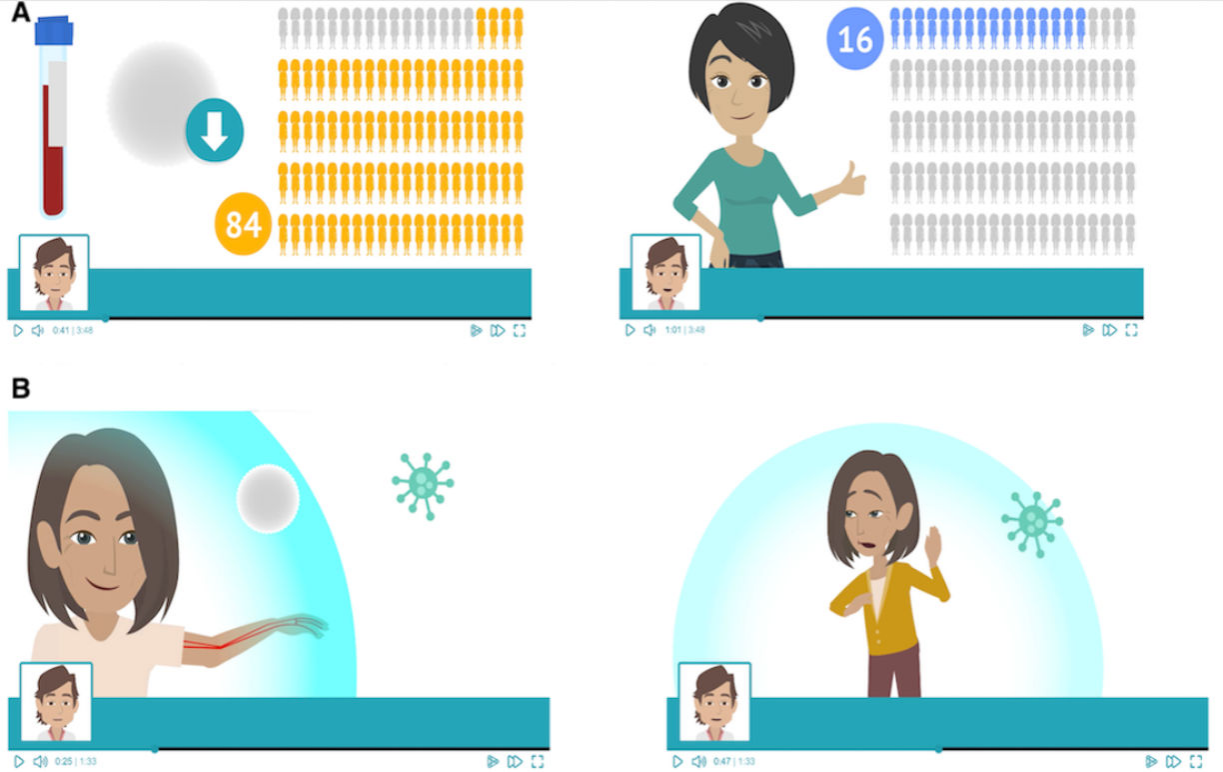
Examples of the presentation of the probabilistic and biomedical information in the tool. (A) Positive and negative framing of probability of experiencing neutropenia. (B) Supporting biomedical information about neutropenia before presentation of outcome data.

**Figure 5.**
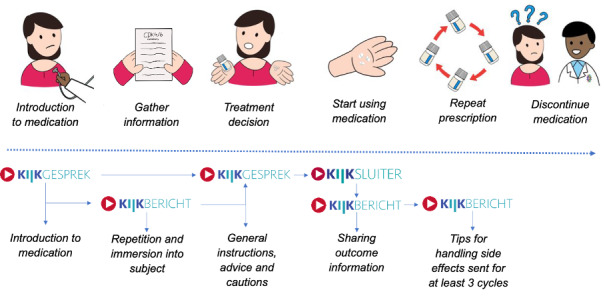
Summary of patient journey including the solutions developed to support information provision at each phase of the patient journey.

**Figure 6.**
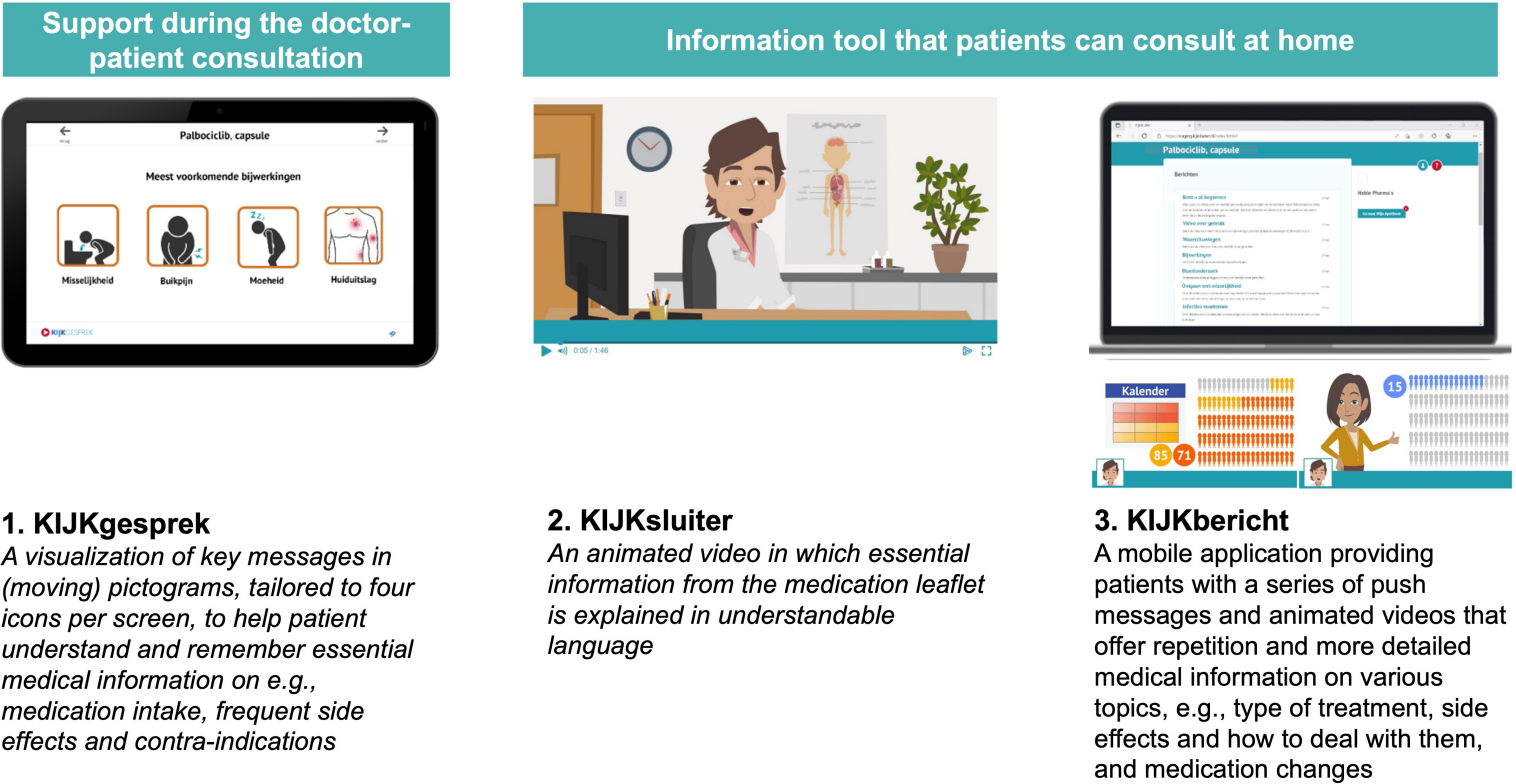
Components of the CDK4/6 inhibitor information tool.

Based on the requirements set by the steering group (using the results from the patient journey mapping and the patient and clinician needs assessment), prototypes for the information tool were developed. Figures 4 and 5 provide a summary of the patient journey, including components of the information tool, to support information provision and treatment decision-making at each phase of the patient journey. The information tool consists of three main components: (1) KIJKgesprek (Stichting Kijksluiter), (2) KIJKsluiter (Stichting Kijksluiter), and (3) KIJKbericht. The 3 components are described in Figure 6. The steering group reviewed the prototypes, and in an iterative process, the prototypes were refined to a version ready for pilot-testing—the high-fidelity prototype.

### Pilot-Testing of the Information Tool Among the Targeted Population

#### Step 1: Status Quo Prior to Implementing the Information Tool

##### Overview

To obtain insight into patients’ experiences with information provision, treatment decision-making, and the treatment burden before the CDK4/6 inhibitor information tool became available, we conducted 20 interviews. Table 3 provides an overview of the participants’ characteristics. We identified 5 key themes and summarized the findings per theme.

**Table 3.** Sociodemographic characteristics of women interviewed during pilot-testing of the information tool.

Characteristics^a^	Step 1: before implementation (n=20), n (%)	Step 2: prototype testing (n=18), n (%)
Marital status		
Married or in a relationship	9 (47)	12 (80)
Single	4 (21)	2 (13)
Widowed	6 (32)	1 (7)
Missing	1	5
Education		
Lower	7 (41)	3 (20)
Intermediate	4 (24)	5 (33)
Higher	6 (35)	7 (47)
Missing	3	5
Occupation		
Paid job	2 (11)	2 (14)
Volunteering	1 (5)	1 (7)
Retired	7 (37)	3 (21)
Unemployed	9 (47)	8 (57)
Missing	1	6
Diagnosis		
Prior breast cancer	13 (93)	10 (67)
Immediate metastasis	1 (7)	5 (33)
Missing	6	5
CDK4/6 inhibitor as:		
First-line	9 (56)	8 (57)
Second- or later-line	7 (44)	6 (43)
Missing	4	6

a In a small number of interviews, certain sociodemographic questions were not asked or probed further because the conversation centered on highly emotional and sensitive topics, and the interviewer prioritized participant comfort and rapport.

##### Patients’ Treatment Knowledge

Most patients could not describe what kind of treatment a CDK4/6 inhibitor is. Many patients mistook the CDK4/6 inhibitor for chemotherapy:

*I understood that the drug is a kind of chemotherapy that fights cancer cells.* [ID03]

Patients did seem to understand that a CDK 4‐6 inhibitor helps to slow or stop the growth of cancer cells. Side effects that most patients knew of before the start of treatment were alopecia, gastrointestinal complaints, fatigue, and hematological changes (eg, neutropenia). Many patients linked blood check-ups to assess how well they were tolerating the treatment and whether they could start a new treatment cycle. There seemed to be variation in information provision; some patients were informed about treatment modification strategies (such as stopping for an additional week before starting the next treatment cycle or lowering the dosage), while others were not.

##### Information Resources Use

Study participants discussed various information resources they consulted, including HCPs, written materials, medication leaflets, and the internet. Patients consulted medication leaflets before or during treatment, finding them helpful but overwhelming due to the extensive list of potential side effects. Many patients preferred information about side effects in smaller doses, and timing of information was also deemed important (eg, getting information when it became relevant for them instead of hearing everything in advance). Some patients sought additional information online, while others avoided it due to concerns about accuracy and biased narratives (eg, overrepresentation of “horror stories”). Although most participants were satisfied with the information they received from HCPs, some felt it was insufficient, particularly regarding the management of side effects. Also, participants felt that the amount of time available to consult with their oncologist was too limited. Overall, participants appreciated that they could contact HCPs for further clarification outside of their scheduled consultations if the need should arise.

##### Patients’ Preferences Regarding Outcome Data

Many participants were receptive to receiving information about potential side effects, with preferences varying in terms of detail. Some preferred information limited to the most common side effects based on real-world data, without probabilities, while others wanted a comprehensive overview including probabilities. Many trusted their oncologist to provide relevant information as needed, expressing no strong desire to receive all information upfront or to use a tool containing such data. For some, not knowing everything served as a coping mechanism to maintain hope and reduce anxiety about factors beyond their control (eg, response to treatment). Despite this, a few participants acknowledged an internal struggle, balancing the desire to understand what could lie ahead with the comfort of remaining uninformed. Additionally, many patients expressed reluctance to receive outcome data related to prognosis, feeling such statistics might not apply to them (eg, “I do not like statistics or prognoses at all, because I always fall outside their scope” [ID01] or “You have to wait and see how things are going to turn out in your situation” [ID02]) and could be too confronting or overly influential on their life choices (eg, “I do not want to think I only have a year left” [ID03]). Some preferred outcome data to be available only upon request or in urgent situations, such as disease progression requiring decision-making. This ambivalence highlights the complex and individualized nature of patients’ preferences for receiving prognostic information.

##### Experienced Treatment Burden

All patients experienced treatment side effects, with about a quarter encountering cycle delay, dose reductions, or interruptions due to, for example, severe side effects. Participants used several coping strategies. Many spoke of acceptance being important to cope with their situation, and acknowledging the futility of questioning what could have been:

*You can start wondering, could I have prevented this [metastatic disease]? Could it have been different? But I know from experience that those kinds of questions don’t get answered anyway, and that’s no use to you. So, you have to make do with what you have at that moment.* [ID04]

Some found comfort in hoping to defy the odds:

*They [clinicians] did say right away, we cannot cure you, but we can make the quality of your life better and prolong your life. But they also said that bit [prolonging life]. So, I’ve had the hope that maybe I would be cured after all...* [ID05]

Denial and trivialization of their symptoms and situation were also common coping mechanisms. Despite many patients rating their quality of life high, many simultaneously acknowledged significant negative impacts on their daily lives due to the disease and/or treatment side effects, and ways they needed to adapt.

##### Treatment Decision-Making

Patients expressed that they felt a sense of urgency to start treatment with a CDK4/6 inhibitor, as it is perceived to be the best available option at this stage of their disease:

*I had already taken letrozole for four years and it wasn’t working anymore […] so you have to switch to something else. For me it feels like I don’t really have a choice […] they know which option is best for me.* [ID02]

A few preferred a CDK4/6 inhibitor due to its perceived lower burden compared with other treatments, such as chemotherapy. Many patients indicated their only other option would be chemotherapy and expressed reluctance to start this, given its potential side effects and impact on their quality of life:

*For me the choice was chemotherapy and its side effects, or this treatment and its side effects. So, I didn’t really focus on what the side effects of this treatment were, because compared with chemotherapy it’s obviously much less severe*. [ID18]

Given that chemotherapy was the alternative, being eligible for treatment with a CDK4/6 inhibitor was seen as positive news. While some patients felt involved in the decision-making process, most patients indicated that their oncologist took the lead in prescribing treatment, often without discussing alternatives. Trust in oncologists played a significant role in patients’ acceptance of treatment decisions. However, some oncologists did offer patients the opportunity to decide or take time to consider their options. Patients commonly cited the desire to extend life, slow cancer cell growth, reduce symptoms, and improve quality of life as motivation for starting CDK4/6 inhibitor treatment.

### Step 2: Prototype Testing

#### Overview

A total of 18 patients pilot-tested the information tool (Table 3). Their experiences with treatment, information provision, knowledge about treatment, and coping strategies aligned with the findings from the women who did not have access to the tool (step 1). Therefore, we only summarize the prototype testing participants’ views and recommendations for each component of the information tool below.

#### Views on the Visualized Key Information for Use During the Clinical Encounter (KIJKgesprek)

Participants generally thought that the so-called KIJKgesprek used during a consultation (a visualization using animated pictograms to help patients understand and remember essential medical information from the consultation, eg, medication intake, common side effects, and contraindications) was well-structured and comprehensive in covering key points. However, opinions varied regarding its added value. While some women believed it could enhance doctor-patient communication by aiding information processing, others doubted its added benefit or expressed concerns about it being potentially distracting to patients (eg, it can be too much to have to look at a screen as well as process what the HCP is saying):

*I didn’t feel it added much value for me, although it might be useful for the oncologist to support the explanation […]. Personally, I didn’t really think it would have added anything to the conversation with my oncologist. Everything was already clear to me. What did stand out to me, though, was that I hadn’t really received the warnings mentioned, such as avoiding St John’s wort, grapefruit, alcohol, and being aware of possible liver problems. That might have been a useful addition.* [ID12]

One participant acknowledged the KIJKgesprek’s potential utility as a guide for HCPs to ensure comprehensive information delivery.

#### Views on the Animated Information Videos

Most participants found the videos informative, and the language use was deemed clear and accessible:

*I found it [information tool] very helpful. I think it can really be a valuable addition when you start with it [treatment] […] although also midway through treatment. I found it clear*. [ID4]

Participants indicated that it would have been of added value to them if they had had access to this tool at the time they started with treatment. While participants believed all relevant topics were covered in the videos, opinions varied about the level of detail. More highly educated participants desired more information and a greater level of detail. The tips for coping with side effects provided in the videos were highly valued by all. Some provided suggestions for more tips that could be added. The videos’ length was deemed appropriate, and participants appreciated the ability to revisit specific topics. Participants saw the tool as a valuable reference, especially for processing information after receiving difficult news. However, a few preferred to avoid excessive information unless necessary, citing concerns about losing hope.

#### Views on the Incorporation of Outcome Data in the Information Tool

Visualization of real-world outcome data in the information tool introduced a novel aspect, but opinions among participants varied. Overall, participants thought that the visualization of the outcome estimates was clear. Some expressed an internal conflict, acknowledging the importance of knowing realistic probabilities of experiencing outcomes while also feeling overwhelmed or wanting to avoid such information:

*It [outcome data] is reality, of course. It’s just that sometimes it depends, it’s good to know, but at times you also don’t want to know. Right?* [ID07]

Many participants believed it was useful to provide patients with realistic outcome data, particularly regarding PFS and side effects. Some participants indicated that they had explicitly requested information about prognosis from their oncologists, and not all had received numerical information. However, many expressed concerns that providing patients with outcome data could diminish hope or discourage treatment initiation (in line with the findings described in the Step 1: Status Quo Prior to Implementing the Information Tool section). Some worried particularly about visualizing the average time the medication is effective in preventing disease progression without adequately acknowledging the variability in treatment response:

*If I had seen before starting [with treatment] that [on average] women only had four months before the disease got worse, I might not have started. For me, that hasn’t been the case. I’m lucky to be able to do well on this [CDK4/6 inhibitor] for years now. … It would be good to explicitly state that the four months is an average, but there are exceptions.* [ID08]

Opinions also diverged on whether to provide outcome data about side effects, with some finding it overwhelming and others valuing the insights and validation of their own experiences:

*Seeing that others [other women using a CDK4/6 inhibitor] also experience the same [complaints] is reassuring, it’s not just me*. [ID09]

### Linking Baseline Patient Experiences to Prototype Evaluation

Overall, step 1 showed substantial gaps in patients’ understanding of CDK4/6 inhibitor therapy, wide variation in information needs and preferences, and challenges in coping with treatment burden and decision-making. These findings underscore the need for structured, accessible, and patient-tailored support materials that incorporate realistic expectations of treatment and side effects. Step 2 demonstrated that the prototype information tool addressed many of these needs. Even though preferences for receiving outcome data varied between patients, many participants did find this information useful. Together, these insights support the incorporation of real-world outcome estimates into the tool.

## Discussion

We present the development and pilot testing of a novel online patient information tool for women with HR_pos_HER2_neg_ metastatic BC undergoing treatment with a CDK4/6 inhibitor. Designed based on patient and HCP needs assessments and input from a multidisciplinary steering group of experts, the tool delivers numerical data on outcomes such as PFS, side effects, and treatment modifications in an accessible format. It also features visualizations to support doctor-patient communication during consultations and includes an app with animation videos and push notifications for home use. By facilitating discussions about treatment outcomes, the tool can help patients form realistic expectations about the benefits and risks of their treatment. This, in turn, is aimed at supporting better-informed SDM and fostering more meaningful patient engagement in their care. Despite its benefits in informing patients and supporting treatment decisions, the use of real-world outcome data in clinical practice is still novel and may be challenging for patients to understand and apply. As its integration becomes more common, it is important to keep in mind that patients may need time to adapt.

Many participants in our pilot test demonstrated significant knowledge gaps regarding CDK4/6 inhibitors. Although participants generally understood that these compounds slow cancer cell growth, awareness of treatment modification strategies and potential side effects was limited. These findings highlight the critical role of patient information tools, like the tool we developed, in promoting consistent and clear communication. Many patients reported that symptom burden significantly impacts their quality of life, underscoring the need for resources with information about strategies to address this challenge [29-31].

Furthermore, challenges in communicating about palliative care often stem from HCPs’ desire to offer hope, which can result in incomplete discussions about treatment options and expected outcomes [32]. The visualizations for use during the consultation incorporated in our tool are designed to support clinicians in delivering complex information more effectively to patients. This approach could be particularly helpful in ensuring patients are better informed, fostering engagement, and setting realistic expectations about their treatment journey. Tailored explanations of treatment mechanisms and side effects, whether detailed or concise, based on individual preferences, can help address misconceptions, reduce concerns, and provide a balanced sense of hope that aligns with patients’ needs and preferences.

Preferences for outcome data among participants in the pilot test were highly individualized. While some patients valued receiving numerical outcome data about side effects and PFS, others preferred to avoid such information to preserve hope or reduce anxiety. Many participants in our pilot test expressed a preference for outcome data to be offered only on request or in specific scenarios, such as if there is disease progression, emphasizing the importance of tailoring and sensitivity in presenting this type of data. These findings align with research showing that patients often prefer to be asked whether they want to receive outcome data, rather than having it provided automatically, and that these preferences may change over time, necessitating periodic reassessment [11]. In this evaluation study, all participants were exposed to the full set of outcome information. However, this does not reflect the intended use of the tool in practice, where information would be shown only at clinically relevant time points. Our findings also support adding advanced notifications (eg, pop-ups), alerting patients that outcome estimates are available. This allows patients to decide whether they wish to view the information, thereby supporting autonomy in how and when they receive potentially sensitive data. Finally, while the tool acknowledges that outcomes are highly individual, we were unable to personalize estimates due to limited available data. Future development could further explore personalized presentations of expected benefits and risks as more real-world data become available.

Participants in the pilot test expressed concerns about the potential for numerical outcome data to diminish hope, particularly when averages were presented without sufficient context regarding variability and uncertainty about outcomes for individual patients. While many patients reviewed the visualizations of outcomes as useful for understanding PFS and side effects, others felt overwhelmed or worried that such information might have deterred them from initiating treatment. This aligns with findings from a recent multicenter survey, which reported that a subset of patients with advanced cancer actively prefers not to receive prognostic information to preserve hope and reduce anxiety [33]. Communicating variability in outcomes using outcome ranges instead of only point estimates and explicitly framing numerical data as averages is especially important in palliative care, where maintaining hope is a central aspect of patient engagement. Balancing honesty about prognosis with empathy is critical for fostering trust and minimizing distress [11]. Striking this balance is essential to align treatment decisions with patients’ values and priorities. There is a need for future research on how negative emotions in SDM may be minimized [34,35].

Some study limitations need to be considered when interpreting the results of this study. Women reviewed all the components of the tool at once, instead of getting the push notifications in line with the phase of treatment they were in, as intended. Consequently, the tool was less attuned to meet women’s diverse informational needs. The benefits of gradual information display and repetition were lost, which may have caused women to feel overwhelmed by (outcome) information. Moreover, the prototype testing phase included more highly educated patients, which may have influenced study outcomes. It requires further testing to determine whether the tools meet the needs of patients with low numeracy and health literacy. Also, the findings of our pilot testing do not provide insight into how the tool will fit into the clinical workflow and potential barriers to implementation. Identifying any potential barriers to clinical use, as well as identifying criteria for when to use and when not to use, is particularly important for the tool component intended for use during consultations (ie, Kijkgesprek). An important strength of this study is the use of real-world outcome data in a patient information tool, specifically to get more realistic data regarding the patient’s experience with side effects and their clinical management. The breadth of patient experiences with treatment side effects captured in the real-world information is more likely to reflect the experiences of patients than information obtained from the highly selected populations in clinical trials. An important strength of the tool design process is the participatory development approach we used, involving both patients and HCPs to ensure the resulting information tool matched their needs and preferences. Finally, given the complexity of communicating and understanding outcome data, great effort has been put into presenting the information in an accessible manner to ensure that patients with low (health) literacy can also understand the information.

In conclusion, we used the context of treatment with a CDK4/6 inhibitor in combination with endocrine therapy for *HR_pos_HER2_neg_* metastatic BC as a case study to assess the value and usability of real-world outcome data in informing patients about their treatment and outcomes. Women in the pilot test rated our tool highly, appreciating its clear and structured information. However, preferences for how to receive (different types of) outcome data varied. To accommodate patients’ varied preferences for outcome data, we recommend that tool developers provide options for patients to choose whether they want to see additional information (eg, survival probabilities) and in how much detail. For instance, using pop-up warnings or expandable text would be beneficial. Moreover, we highly encourage future studies to evaluate the usage of our tool in clinical practice. In our study, we identified the facilitators and barriers that need to be addressed to enable the successful (nationwide) implementation of our information tool. Finally, our study can serve as a template for others who want to develop patient information tools incorporating real-world data. As most patients are open to receiving outcome data, concerted efforts should be made to set up the infrastructure to collect the necessary data and facilitate its effective use in patient information tools.

## Supplementary material

10.2196/73156 Multimedia Appendix 1 Interview guide.

10.2196/73156 Multimedia Appendix 2 Steering group members.

## Abbreviations

BC: breast cancer
HCP: health care professional
HER2_neg_: human epidermal growth factor receptor 2–negative
HR_pos_: hormone receptor–positive
PFS: progression-free survival
SDM: shared decision-making
VBHC: value-based health care
