# Supplementary material for: An Information Tool Incorporating Real-World Outcome Data for Women With Metastatic Breast Cancer Eligible for Treatment With a CDK4/6 Inhibitor: Development and Evaluation
Source: JMIR Cancer. 2026 Jul 30;12:e73156. doi: 10.2196/73156 (PMC13420873; doi:10.2196/73156)
Supplement: Multimedia Appendix 1 [file cancer-v12-e73156-s001.docx]

**Appendix A. Interview guide**

Part 1: Introduction

[Interviewer introduces themselves and gives a brief introduction about Santeon. Mention that you are not a doctor and therefore cannot answer medical questions.]

Santeon introduction

Santeon is a group of seven top clinical hospitals working together to improve medical care through continuous innovation. By observing each other’s practices, these hospitals can learn from each other and continuously improve patient care.

Briefly reiterate the research objective and explain the interview procedure.

The outcomes of care and the experiences of other patients in similar situations can help patients make treatment decisions. Think of information such as: how effective is the treatment and what side effects occur. With this interview, we aim to find out whether providing information about such care outcomes makes patients feel better informed and more involved in decision making about treatment with a CDK4/6 inhibitor. The information we gather from these interviews will help us identify the strengths of the current information provision and what can be improved. Do you have any questions at this moment?

Ask for permission to record and start the audio device

In the consent form, you gave permission to record this interview, so it can be transcribed and analyzed. The recording will be destroyed after transcription. The texts will be processed anonymously, without names. Transcription of the recording will be done by an external agency, as mentioned in the patient information you received. You also gave permission for this. Do you agree that I start the recording now?

[Start recording]

Part 2: Clinical background, understanding of treatment and preferences and experience with information provision

1. To start, I want to ask, how are you doing?
2. Can you briefly describe your treatment journey so far?
3. Can you tell us about the process of deciding whether or not to start treatment with palbociclib?
   1. Prompt: What prompted the start of using palbociclib?
   2. Prompt: Who explained to you that using palbociclib was an option for you?
   3. Prompt: To what extent did you use digital resources to gather information about palbociclib? Which websites did you visit?
4. How have you experienced using palbociclib so far? (only if patient already started using palbociclib)
5. The next question is about how you perceive your quality of life. How would you rate your overall "quality of life" during the past week? (7-point scale; 1= very poor, 7= excellent) (probe if the past week is a good reflection of overall quality of life)
6. How did you find the information provided about palbociclib?

To make a decision about treatment with palbociclib, it might help to have information about how previous patients have fared. The following questions are about how you look back on the information you received and how important you find various topics.

Note regarding the approach for questions 7 through 9:

- Start with a general question: "Do you feel sufficiently informed about the side effects that patients might experience?" and "How important is it for you to hear this information before starting treatment?"
- Then a general question about the need for outcome data: "Would it be valuable for you to hear statistics about how many women experience certain side-effects?"
  - Depending on the answer:
    - If no need: check if this is only at the beginning of the process. Would such information be valuable as a reference, e.g., to see if certain complaints are normal?
    - If yes: then fully explore follow-up questions.

1. Have you received enough information about the treatment with the drug palbociclib regarding...

... the side-effects that patients experience?

... the extent to which patients experience side-effects?

... advice from patients on how to manage side-effects?

... the extent to which patients are prescribed a dose interruption* during treatment? (*if unfamiliar with the term, explain)

... the extent to which patients are prescribed an extended stop week during treatment?

... the extent to which patients are prescribed a dose reduction during treatment?

... how long patients can use the treatment on average?

... further treatments that patients have undergone after palbociclib?

... the risk of death from advanced or metastatic breast cancer?

1. How important is information about...

... the side-effects that patients experience?

... the extent to which patients experience side-effects?

... advice from patients on managing side-effects?

... the extent to which patients are prescribed a dose interruption during treatment?

... the extent to which patients are prescribed an extended stop week during treatment?

... the extent to which patients are prescribed a dose reduction during treatment?

... how long patients can use the treatment on average?

... further treatments that patients have undergone after palbociclib?

... the risk of death from advanced or metastatic breast cancer?

1. Broad starter question: To what extent would information about how previous patients have fared with this treatment help you in making a decision about starting the treatment? [ask the question openly without categories and give the participant space to answer, then check which category the participant would choose]
   1. Prompt: To what extent would information about the side-effects experienced by patients previously treated with palbociclib help you in making a decision about starting the treatment? [ask the question openly without categories and give the participant space to answer, then check which category the participant would choose]
2. Have you received information about this from your healthcare provider? If yes: Can you tell us about it?
3. Have you read information about side-effects in the package insert?
4. Have you looked up information yourself? If yes: Where did you find information? Can you tell us about the information you found?
   1. Prompt: To what extent would information about the life expectancy of patients previously treated with palbociclib help you in making a decision about starting the treatment?
      1. Have you received information about this from your healthcare provider?
         - If yes: Can you tell us about it? What did you think about this being discussed during your conversation with your healthcare provider?
         - If no: Would you have wanted this information in your conversation with your healthcare provider? Why or why not?
      2. Have you looked up information yourself? If yes: Where did you find information? Can you tell us about the information you found?
5. If you had to express your satisfaction with the information provision in a number, what number would you give? (10-point scale; 1= very poor, 10= excellent)

Your healthcare providers aim to inform you as fully as possible about the potential pros and cons of the treatment they propose. This information can sometimes be difficult to understand. Next, I will ask you to describe the treatment with palbociclib in your own words. It is not intended as a test, but we want to know how well the information is provided and where we might need to pay more attention.

1. Can you describe in your own words what the purpose of treatment with palbociclib is?
   - - - Prompt: What kind of drug is palbociclib?
       - Prompt: Can you describe in your own words what palbociclib does?
       - Prompt: Is it an option not to undergo the treatment?
2. Can you indicate which side-effects the treatment with palbociclib can cause?
   - - - Prompt: Have you received information about what you can do if you experience certain side effects? If yes, can you tell us about it?
       - Prompt: Have you received information about which symptoms require contacting the hospital? If yes, can you tell us about it?
3. Why is blood frequently drawn during treatment with palbociclib?
   - - - Prompt: Have you received information about what the doctor looks for in the blood? If yes, can you tell us about it?
       - Prompt: Have you received information about what it means for continuing treatment if the blood values are "not good"? If yes, can you tell us about it?
4. Is there any information about treatment with palbociclib that you missed or found unclear?

Part 3: Satisfaction with the information tool (only for participants in part 2 who had access to the tool)

1. What did you think of the information tool?
   1. Did the information tool help you in understanding the treatment with palbociclib?
   2. Was the information in the tool clear and understandable?
   3. Was the information in the tool relevant to you?
2. Do you have suggestions for improving the information tool?
   1. Did you miss any topics?
   2. Are there topics you think should be removed?

Part 4: Closing

We have now reached the end of the interview. We have discussed a lot, but do you have any additional comments? Thank you very much for your participation and the time you took for this interview. The insights you provided will help us improve our information provision.

[Stop recording]
