# Supplementary material for: An Information Tool Incorporating Real-World Outcome Data for Women With Metastatic Breast Cancer Eligible for Treatment With a CDK4/6 Inhibitor: Development and Evaluation
Source: JMIR Cancer. 2026 Jul 30;12:e73156. doi: 10.2196/73156 (PMC13420873; doi:10.2196/73156)
Supplement: Multimedia Appendix 2 [file cancer-v12-e73156-s002.docx]

**Appendix B. Steering group members.**

Initiators:

- M.Q.N. Hackert, postdoctoral researcher at Santeon
- C.F. van Uden-Kraan, program manager at Santeon
- E.M.W. van de Garde, hospital pharmacist at St. Antonius Hospital and associate professor of clinical pharmacotherapy at Utrecht University

Additional members:

- A. Denis, patient representative at the Dutch Breast Cancer Society
- E. Kooijmans, patient representative at the Dutch Breast Cancer Society
- M. Velting, patient advocate at the Dutch Breast Cancer Society
- M.J. Agterof, internist-oncologist at St. Antonius Hospital
- A.W.G. van der Velden, internist-oncologist at Martini Hospital
- C. Putker-Hartzmann, nurse specialist at St. Antonius Hospital
- K. Godee, nurse specialist at OLVG
- E. Bolt, oncology nurse at Martini Hospital
- J. Bakker, information architect at Catharina Hospital
- M. de Weerd, health information consultant at Canisius-Wilhelmina Hospital

Facilitators of the development process:

- I. Coenraad, director Patient Journey Lab
- A. de Jong, chief operations officer and co-founder CareAnimations
- R. Neeter, chief executive officer and co-founder CareAnimations
- S. Eising, director Stichting Kijksluiter
- A. Vogelaar, project manager at Santeon
- E.G. Engelhardt, postdoctoral researcher at Santeon

When additional expertise was needed, additional stakeholders in the Santeon hospitals were consulted.
